# Preliminary evaluation of the German SPIN and Mini-SPIN for screening social anxiety disorder in university students

**DOI:** 10.1186/s40359-026-05119-8

**Published:** 2026-07-10

**Authors:** Teresa Schmidt-Peter, Leon O. H. Kroczek, Andreas Mühlberger

**Affiliations:** Department of Psychology, Clinical Psychology and Psychotherapy, University of Regensburg, Regensburg, Germany

**Keywords:** Social anxiety disorder, Social phobia, Screening, Validity, Diagnostic interview

## Abstract

**Background:**

Efficient screening for Social Anxiety Disorder (SAD) requires valid and up-to-date instruments. The Social Phobia Inventory (SPIN) is a widely used and internationally validated measure. The German version has been validated in clinical and community samples, with a cut-off score of 25 established in 2008. However, changes in social interactions and communication patterns may influence responses to items, particularly in student populations. Moreover, the original German validation relied on DSM-IV criteria, and its validity under DSM-5 has not yet been sufficiently investigated. Given the increasing demand for brief screening tools, the Mini-SPIN offers a time-efficient alternative; however, its diagnostic accuracy in student populations has not been systematically examined.

**Method:**

In this preliminary study, 65 non-treatment-seeking German psychology students completed the SPIN, Mini-SPIN, and a structured clinical interview for SAD based on DSM-5 criteria. Receiver operating characteristic analyses were conducted to assess discriminative accuracy and determine optimal cut-off scores.

**Results:**

The SPIN showed moderate discriminative accuracy in distinguishing students with and without SAD using the established cut-off score of 25, yielding a sensitivity of 80%, a specificity of 69%, and an area under the curve of 0.71. The Mini-SPIN showed lower discriminative accuracy in this sample, suggesting that the full SPIN may be the more suitable screening instrument for comparable populations. Exploratory analyses suggested different optimal cut-off scores for female and male participants, warranting replication in larger samples.

**Conclusion:**

The findings provide preliminary support for the continued applicability of the SPIN as a screening tool in contemporary student populations. Incorporating brief assessments of distress and functional impairment may further improve screening accuracy. Further research should examine sex-specific cut-off scores in larger and more diverse samples.

**Supplementary Information:**

The online version contains supplementary material available at 10.1186/s40359-026-05119-8.

## Introduction

Social Anxiety Disorder (SAD), also known as Social Phobia, is a prevalent and impairing mental disorder characterized by an intense and persistent fear of negative evaluation or of being scrutinized by others in social and/or performance situations. The disorder is typically accompanied by pronounced avoidance behaviors and leads to severe impairments across social, academic, and occupational domains [2].

SAD typically emerges in late childhood or adolescence and affects a considerable proportion of the population, with lifetime prevalence rates ranging from 6% to 13% in European and U.S. samples [9, 13]. When the disorder remains undetected or untreated, SAD is associated with a high risk of chronicity and long-term functional impairment [9].

Despite its high prevalence and significant burden of disease, SAD frequently remains undetected in routine clinical and academic settings. This is partly due to symptom overlap with other anxiety disorders, depressive conditions, and substance abuse as well as to the tendency of individuals with SAD to avoid help-seeking [9]. Although social fears are common in the general population, not all individuals experiencing fear in social situations meet diagnostic criteria for SAD. Consequently, efficient, reliable, and time-saving screening instruments are essential for identifying clinically relevant social anxiety.

The Social Phobia Inventory (SPIN; [6] is a widely used self-report questionnaire for screening SAD and is often regarded as a gold standard screening instrument in both research and clinical practice, particularly in German-speaking contexts [21]. The SPIN consists of 17 items and assesses core dimensions of SAD, including fear, avoidance, and physiological distress, and has demonstrated robust psychometric properties across clinical, community, and student samples. In the original validation study, Connor et al. [6] proposed a cut-off score of 19 to distinguish individuals with SAD from non-anxious controls in clinical samples. The German version of the SPIN was translated by Stangier & Steffens [22] and validated in representative and clinical samples by Sosic et al. [20]. In contrast to Connor et al. [6], the German validation by Sosic et al. [20], based on both a representative population and a clinical validation sample, suggested a higher cut-off score of 25 to optimize sensitivity (85%) and specificity (68%). Interestingly, although [20] Study 1) reported gender differences in SPIN scores in a general population sample, gender-specific analyses were not presented in their validation study (Study 2), and a single overall cut-off score was proposed, possibly due to limited sample sizes in the clinical subsamples. Overall, the SPIN has demonstrated good reliability, validity, and diagnostic accuracy across different populations (e.g., [6, 17, 21]. 

Importantly, these validation studies and the derived cut-off recommendations were established more than a decade ago based on DSM-IV diagnostic criteria and under social and cultural conditions that differ from those experienced by contemporary student populations. Changes in social interaction patterns, including the increasing relevance of digital communication and evolving social norms, may influence how social anxiety symptoms are expressed and reported, as online social environments introduce novel forms of social evaluation, comparison, and feedback (e.g., [1, 15]). Contemporary student populations may be particularly relevant for examining such developments, as young adults are highly exposed to digital communication, online social comparison, and evolving social interaction norms. Furthermore, recent studies indicate an increase in social anxiety symptoms, particularly among adolescents and young adults, with consistently higher prevalence and greater increases in rates reported for women [4, 8, 18]. Overall, these developments highlight the importance of examining whether previously established SPIN cut-off values remain applicable in contemporary student samples under DSM-5 diagnostic criteria, and whether potential sex-related differences suggested in earlier studies (e.g., [20]) warrant further investigation.

In addition to the SPIN, the Mini-Social Phobia Inventory (Mini-SPIN; [7]), which consists of three selected items from the full SPIN, was developed as a highly economical screening instrument. The German Mini-SPIN was validated in clinical and representative community samples, with a recommended cut-off score of 6 for probable SAD [24]. Although its brevity and simplicity make it attractive for rapid screening in research and nonclinical settings, its diagnostic accuracy has not yet been systematically examined in contemporary student populations.

Based on the considerations outlined above, the present preliminary study aims to evaluate the diagnostic accuracy of the SPIN and the Mini-SPIN in a contemporary sample of German psychology students. The primary objective is to examine whether the SPIN still represents a valid screening instrument for social anxiety in this population, with particular focus on the established cut-off score of 25. Both SPIN and Mini-SPIN are compared against the SCID-5-CV (DSM-5), the current diagnostic gold standard. Sex-related differences are explored descriptively. In addition, the Mini-SPIN is examined in an exploratory manner with regard to its current suitability and optimal cut-off for rapid screening.

Despite societal changes and altered patterns of social interaction, we hypothesize that the SPIN will demonstrate moderate to high discriminative accuracy in the present sample, corresponding to an area under the ROC curve (AUC) of at least 0.70, in distinguishing participants with and without SAD diagnosis.

## Method

The present study represents a standalone analysis of data collected within a larger preregistered project on virtual reality-based presentation training (AsPredicted #198,252). While the main study focused on outcomes of public speaking training, the current analysis specifically addresses the psychometric validation of the SPIN and Mini-SPIN questionnaires against the SCID-5-CV and the determination of optimal cut-off scores in a university student sample. Procedures, measures, and results related to the broader virtual reality training are not reported here; this paper focuses exclusively on the evaluation of these questionnaires as screening instruments for SAD.

### Participants

Participants were non-treatment-seeking undergraduate psychology students (≥ 18 years and German language proficiency of at least B2) enrolled in a course at the University of Regensburg. This course is part of the B.Sc. Psychology curriculum and is typically taken in the third semester. Participation was voluntary, and sample size was determined by the course framework and available resources.

Exclusion criteria included self-reported significant neurological disorders like epilepsy, current or past psychotic symptoms (e.g., delusions, hallucinations), vestibular or balance impairments, pregnancy, and other severe physical or mental limitations that could interfere with study participation, especially experiencing a virtual reality session.

The final sample consisted of 65 participants (mean age = 21.02 years, *SD* = 2.74, range 18–32 years), including 58 females (89.2%) and 7 males (10.8%). The highest level of education attained was a general qualification for university entrance (90.6%), followed by a university degree (6.3%) and vocational training (3.1%). Full demographic information is provided in Supplementary Table S1.

Participants could choose between course credit or monetary compensation of 15 EUR. Study procedures were approved by the Ethics Committee of the University of Regensburg (reference number 20–2086-10). All participants provided written informed consent after receiving detailed information about study procedures.

### Measures

#### Demographic questionnaire

Participants completed a short self-constructed questionnaire assessing demographic data (age, gender) and inclusion/exclusion criteria (German language proficiency, psychotic symptoms, seizure or neurological disorders, organic dizziness, gait or balance problems, pregnancy, and severe physical impairments) via self-report. The questionnaire was administered online via SoSci Survey (Version 3.7.06; Leiner, 2025, Munich, Germany).

#### Structured clinical interview for DSM-5 disorders (SCID-5-CV; [10])

The Structured Clinical Interview for DSM-5 Disorders, Clinician Version (SCID-5-CV), is a semi-structured diagnostic interview designed to facilitate reliable DSM-5 diagnoses. It consists of standardized questions organized into disorder-specific modules. In the present study, only the module assessing Social Anxiety Disorder was administered to determine whether participants met DSM-5 diagnostic criteria for SAD. The German version of the SCID-5-CV [5] was used.

#### Social phobia inventory (SPIN; [6])

The SPIN consists of 17 items assessing fear, avoidance, and physiological symptoms of social anxiety during the past week. Each item is rated on a 5-point Likert scale (0 = not at all to 4 = extremely), yielding a total score from 0 to 68. The SPIN has demonstrated good psychometric properties, including internal consistencies between α =.82 - 94 and 2-week test-retest reliabilities between *r* =.78 -.89 [6]. In this study, the validated German version by [22] was used. The commonly recommended German cut-off of 25 [20] was examined and compared against SCID-5-CV diagnoses in this sample.

### Procedure

Only the procedures relevant to the present study are reported here. The overall study procedure was largely based on the design described by [14], with adaptation specific to the present research questions. Data collection took place across five undergraduate psychology seminars at the University of Regensburg, with 105 students enrolled across all seminars, of which 65 participated in the current study. After a brief in-class introduction to the study, students who agreed to be contacted by the research team received an email with a link to an online screening survey (SoSci Survey).

At the beginning of the survey, participants were informed about the study objectives, procedures, and the approximate duration, and provided informed consent. Demographic information (age, gender, educational level) and health-related self-report items were collected to assess eligibility.

Participants who met all inclusion criteria were invited to an experimental session. At the beginning of this appointment, social anxiety was assessed using the SPIN [22] and the SAD module of the SCID-5-CV [5]. All interviews were conducted by advanced master’s students in psychology with a focus on clinical psychology and psychotherapy, lasting approximately 20 min. To minimize potential diagnostic bias, both the interviewers and the master-level psychologist (psychological psychotherapist in advanced CBT training) responsible for reviewing the diagnostic classifications were blind to participants’ SPIN and Mini-SPIN scores during the diagnostic interview and the initial diagnostic classification. Participants with a positive SAD diagnosis were offered optional feedback, including information about their assessment results, available treatment options, and general advice for seeking professional support. After completion of the SCID-5-CV module, the part of the study relevant to the present analysis was concluded.

### Statitical analysis

All statistical analyses were conducted using SPSS Statistics (Version 29.0.0.0; SPSS INC., Chicago, IL). Descriptive statistics (means, standard deviations, and ranges) were calculated for both SPIN and Mini-SPIN total scores for the full sample and separately by sex. The Mini-SPIN score was computed for each participant using the sum of the three SPIN items (items 6, 9, and 15) comprising the Mini-SPIN [7, 24].

To evaluate the diagnostic performance of the SPIN and Mini-SPIN, receiver operating characteristic (ROC) analyses were performed with the SCID-5-CV diagnosis as the criterion variable. From the ROC curves, sensitivity, specificity, and the area under the curve (AUC) were derived. The Youden Index (J = sensitivity + specificity − 1) was calculated, and the optimal cut-off value for both instruments was determined at the maximum Youden Index.

Descriptive comparisons of scores between sexes were conducted for exploratory purposes, given the small number of male participants. The significance level was set at α = 0.05.

## Results

### Descriptive statistics and diagnostic status

**Fig. 1:**
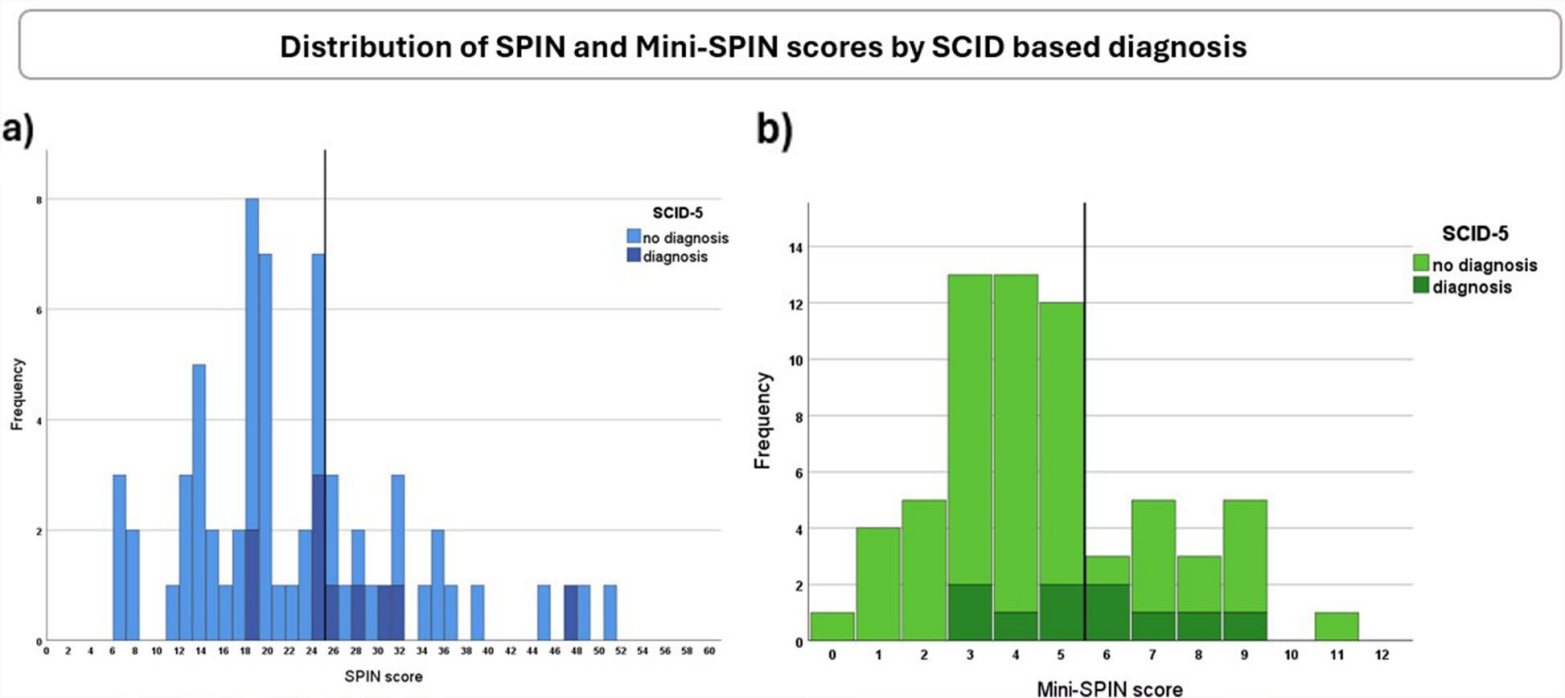
Histogram depicting the frequency of SPIN scores (**a**) and Mini-SPIN scores (**b**) among all participants (*N* = 65). Bars represent the total number of participants per score and are displayed as stacked bars. Colors indicate the instrument (SPIN = blue, Mini-SPIN = green), and color intensity indicates SCID-based diagnosis (dark = presence, light = absence of social anxiety disorder). In (**a**), the vertical black line represents the established cut-off score of 25 [20], and in (**b**), the established cut-off score of 6 [24]

SPIN total scores ranged from 7 to 51 (possible range: 0–68), with a mean of *M* = 22.66 (*SD* = 9.98). The SPIN demonstrated good internal consistency in the present sample (Cronbach’s α = 0.89). Mini-SPIN scores ranged from 0 to 11 (possible range: 0–12), with a mean of *M* = 4.58 (*SD* = 2.35). Internal consistency of the Mini-SPIN was moderate (Cronbach’s α = 0.64). According to SCID-5, 10 participants (15.4%) met diagnostic criteria for SAD, whereas 55 (84.6%) did not. Descriptive statistics for the total sample and stratified by sex are presented in Table 1. Total score distributions for the SPIN and Mini-SPIN by SCID-based diagnosis are presented in Fig. 1, with additional sex-specific histograms provided in Supplementary Figure S1.

**Table 1:** Descriptive statistics for SPIN, Mini-SPIN, and SAD diagnosis in the total sample and by sex

	**Total** (N = 65)	**Female** (n = 58)	**Male** (n = 7)
**SPIN**, *M (SD)*			
*Total score*	22.66 (9.98)	23.84 (9.83)	12.86 (4.22)
*Fear*	7.98 (4.10)	8.51 (3.99)	3.57 (1.40)
*Avoidance*	8.94 (4.32)	9.36 (4.29)	5.43 (2.82)
*Physiological*	5.74 (2.71)	5.97 (2.73)	3.86 (1.67)
**Mini-SPIN**, *M (SD)*			
*Total score*	4.58 (2.35)	4.84 (2.33)	2.43 (1.13)
**SAD diagnosis**, *n* (%)	10 (15.4)	9 (15.5)	1 (14.3)

SPIN = Social Phobia Inventory (possible range: 0–68) with its subscales: Fear (6 items), Avoidance (7 items), and Physiological (4 items); Mini-SPIN = abbreviated version of the SPIN (possible range: 0–12); SAD = Social Anxiety Disorder assessed with the Structured Clinical Interview for DSM-5-Clinician Version (SCID-5-CV). Values are presented as means (*M*) and standard deviations (*SD*) unless otherwise indicated. Sex refers to biological sex (female/male)

### Diagnostic accuracy

#### SPIN

Using the SPIN cut-off of 25, sensitivity was 80%, specificity was 69.1%, the positive predictive value (PPV) was 32%, and the negative predictive value (NPV) was 95%. Corresponding cross-tabulations are presented in Table 2. Sex-stratified cross-tabulations are provided in Supplementary Table S2.

**Table 2:** Cross-tabulation of SPIN-based classification (cut-off ≥ 25), Mini-SPIN based classification (cut-off ≥ 6) and SCID-5 diagnosis of SAD

		**SCID-5**	
**SPIN ≥ 25**^a^		*criteria fulfilled*	*criteria not fulfilled*
	*cut-off fulfilled*	TP = 8 (12.3%)	FP = 17 (26.2%)
	*cut-off not fulfilled*	FN = 2 (3.1%)	TN = 38 (58.5%)
		**SCID-5**	
**Mini- SPIN ≥ 6**^b^		*criteria fulfilled*	*criteria not fulfilled*
	*cut-off fulfilled*	TP = 5 (7.6%)	FP = 12 (18.6%)
	*cut-off not fulfilled*	FN = 5 (7.6%)	TN = 38 (66.2%)

*TP* True positive, *FP* False positive, *TN* True negative, *FN* False negative, *SPIN* Social Phobia inventory, *SCID-5* Structured Clinical Interview for DSM-5

^a^The table displays the frequency and corresponding percentages (in %) of individuals classified as having or not having SAD according to SPIN and SCID-5

^b^The table displays the frequency and corresponding percentages (in %) of individuals classified as having or not having SAD according to Mini-SPIN and SCID-5. Frequencies and percentages of concordant (TP, TN), and discordant (FP, FN) classifications are presented

**Fig. 2:**
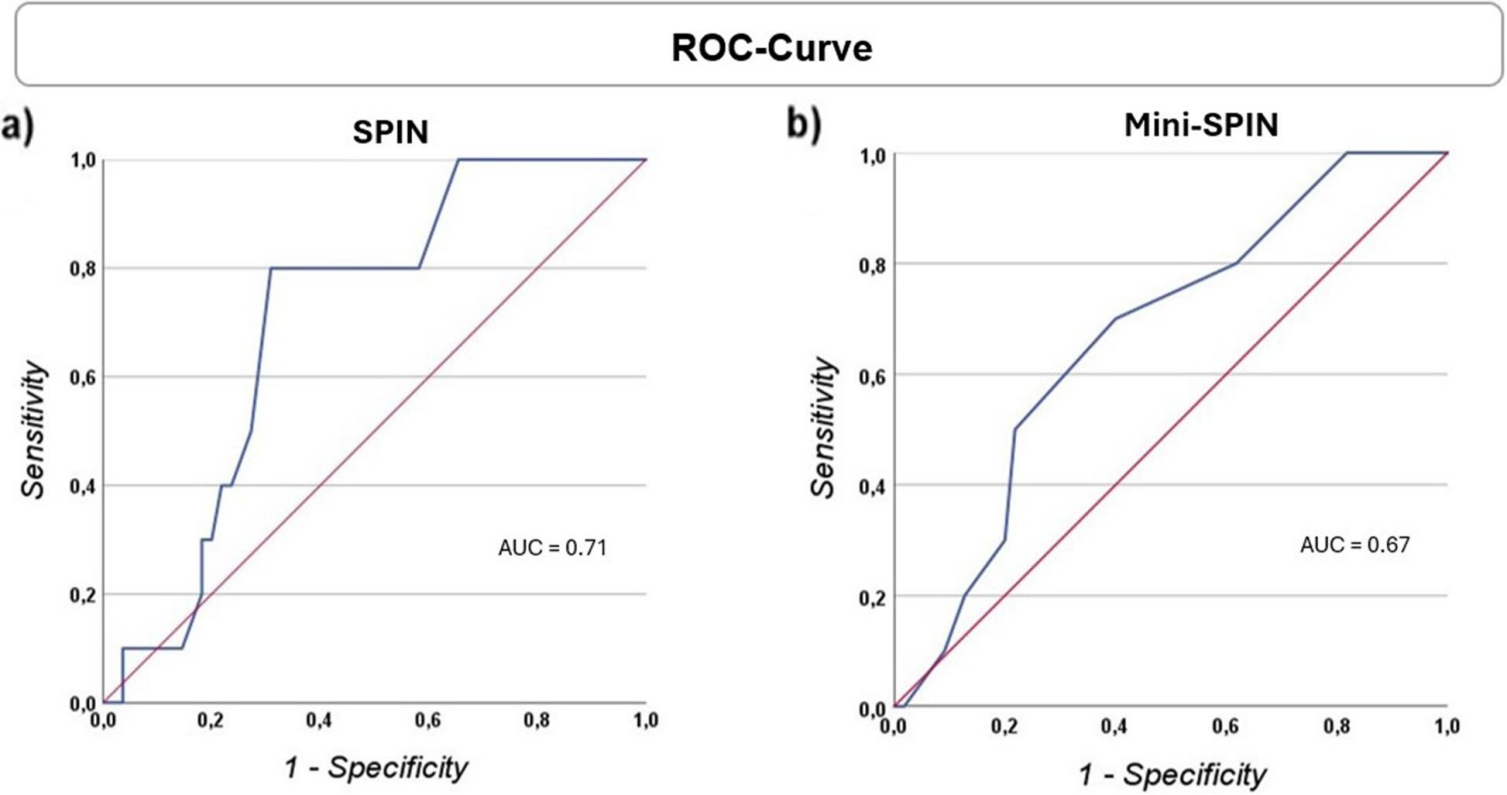
Receiver Operating Characteristic (ROC) curve of the SPIN (**a**) and the Mini-SPIN (**b**) for distinguishing between SCID-positive and SCID-negative cases. **(a)** The area under the curve (AUC = 0.705, *p* = 0.041) indicates moderate discriminative accuracy, above chance level (see red diagonal reference line). **(b)** The area under the curve (AUC = 0.669, *p* = 0.091) indicates low to moderate discriminative accuracy, above chance level (see red diagonal reference line)

A ROC analysis was conducted to assess the diagnostic accuracy of the SPIN in the present sample (Fig. 2a). The area under the curve (AUC) was 0.705 (95% CI [0.561, 0.848], *p* = 0.041), indicating that the SPIN had a moderate ability to discriminate between participants with and without SCID-5 diagnoses. The AUC was tested against chance level (AUC = 0.5). Using the Youden index, the optimal cut-off for this sample was 24.5, resulting in a sensitivity of 80% and a specificity of 69.1%, closely aligning with the recommended cut-off of 25. Exploratory sex-specific analyses suggested an optimal cut-off of 24.5 for females and 18 for males. Given the small and unbalanced subsample, these estimates should be considered with caution and are reported in Supplementary Table S3.

#### Mini-SPIN

Applying the Mini-SPIN cut-off of 6, sensitivity was 50% and specificity was 78.2%, with a PPV of 29.4% and an NPV of 89.6%. Corresponding cross-tabulations are presented in Table 2. Sex-stratified cross-tabulations are provided in Supplementary Table S2.

To further examine the instrument’s diagnostic accuracy, a ROC analysis was conducted (Fig. 2b). The area under the curve (AUC) was 0.669 (95% CI [0.503, 0.835], *p* = 0.091). Although the ROC curve lies above the diagonal reference line representing chance level, the confidence interval includes values near 0.5 and the AUC was not statistically significant, indicating that the Mini-SPIN’s ability to discriminate between participants with and without SAD diagnoses cannot be reliably distinguished from chance in this sample. The AUC was tested against chance level (AUC = 0.5). Based on the Youden index, the optimal cut-off for this sample was 4.5, resulting in a sensitivity of 70% and a specificity of 60%. Exploratory sex-specific analyses suggested an optimal cut-off of 4.5 for females and 3 for males. Given the small and unbalanced subsample, these estimates should be considered with great caution and are reported in Supplementary Table S3.

## Discussion

The aim of the present study was to provide a preliminary evaluation of the diagnostic accuracy of the SPIN in a contemporary sample of German psychology students and to examine whether the established German cut-off value remains applicable under DSM-5 diagnostic criteria. Despite societal changes since the last German validation study [20], including the COVID-19 pandemic, increased digitalization, and evolving social interaction patterns, the present findings suggest that the established cut-off of 25 showed moderate discriminative accuracy in distinguishing students with and without SAD. The optimal cut-off identified in the present sample (24.5) was nearly identical to the original recommendation, and sensitivity and specificity values were comparable to those reported in previous validation studies [20, 21], thereby providing preliminary support for the continued applicability of the established German SPIN cut-off and representing a preliminary replication of the findings reported by Sosic et al. [20] in a contemporary student sample assessed according to DSM-5 criteria.

Notably, several participants exhibited elevated SPIN scores (up to 50) despite not meeting diagnostic criteria for SAD according to the SCID-5-CV. In these instances, diagnostic criteria were not fulfilled because participants did not report clinically significant distress or functional impairment in social or occupational domains during the SCID interview. While the SPIN effectively captures the frequency and intensity of social anxiety symptoms, it does not explicitly assess the criterion of clinically significant distress or impairment required for a SAD diagnosis. This discrepancy illustrates a fundamental distinction between symptom-focused self-report screening instruments and structured diagnostic interviews, highlighting the necessity of considering subjective distress and functional impairment when interpreting elevated SPIN scores. Future iterations of the SPIN may benefit from the incorporation of one or two items explicitly evaluating clinically significant distress or functional impairment, analogous to the SCID-5 diagnostic criterion, to enable more precise clinical interpretation of high screening scores.

Beyond this conceptual distinction, previous research has identified psychometric limitations of the SPIN, particularly concerning the physiological arousal subscale [3, 21]. Factor-analytic investigations indicate that this subscale contributes less uniquely to the assessment of social anxiety, potentially due to its limited number of items and heterogeneous content [3]. In the present study, certain physiological anxiety symptoms reported during the SCID-5 interview were not fully captured by the SPIN items. Although physiological symptoms are not required for a SAD diagnosis, refinement of this subscale could enhance the descriptive precision of the instrument without compromising its primary screening utility.

The Mini-SPIN was examined exploratorily due to its utilization as a rapid screening instrument (e.g., [7, 11, 24]. Although it demonstrated some ability to differentiate between participants with and without SAD, its discriminative performance was markedly inferior to that of the full SPIN in the present sample. Notably, the AUC did not differ significantly from chance, and the established cut-off of 6 showed limited diagnostic accuracy in the present sample. The comparatively low internal consistency of the Mini-SPIN (α = 0.64) should be interpreted in light of its very brief three-item format, as lower reliability coefficients are commonly observed in short screening instruments [23]. Given these findings and the small sample size, the Mini-SPIN should be employed with caution in comparable student populations, whereas the full SPIN remains both efficient and diagnostically superior.

Exploratory analyses based on participants’ biological sex revealed patterns broadly consistent with previous research reporting higher prevalence and greater impairment associated with social anxiety in women (e.g., [8, 16]). Moreover, exploratory analyses yielded different optimal cut-off values for female and male participants, consistent with approaches used for other screening instruments such as the AUDIT [19]. However, the markedly unbalanced sex distribution in the present sample, particularly the very small number of male participants (*n* = 7), substantially limits interpretability. Consequently, these findings should be considered highly preliminary and interpreted with great caution. Importantly, the present data do not provide sufficient evidence to support sex-specific cut-off recommendations. The observed differences are consistent with earlier evidence suggesting gender-related differences in overall SPIN scores ([20] Study 1). However, future studies with larger and more balanced samples are needed to determine whether sex- or gender-specific cut-off values are clinically meaningful and improve screening accuracy.

Beyond these considerations, the present findings should be interpreted considering the specific characteristics of the sample. Participants were non-treatment-seeking psychology students within a relatively narrow age range (18–32 years, *M* = 21.02). University students represent a population of particular interest for the present research question, given the increasing relevance of digital communication and evolving social interaction patterns discussed above. At the same time, these characteristics limit the generalizability of the findings to adolescents, older adults, clinical or treatment-seeking samples, and non-student populations. Furthermore, only ten participants met diagnostic criteria for SAD, which may have reduced the precision and stability of the ROC-based estimates. Future studies should therefore seek to replicate these findings in larger and more diverse samples.

Finally, social interaction contexts have evolved substantially since the SPIN was first developed, particularly with regard to online communication and digital evaluation [12, 26]. Future research should consider updating or supplementing existing items to capture contemporary manifestations of social anxiety, such as the fear of negative evaluation in online settings [25]. Furthermore, the sensitivity of the German SPIN to therapeutic change has not yet been systematically evaluated and represents a further critical avenue for future investigation. Despite these limitations, the present findings provide preliminary support for the SPIN as a screening tool in comparable student populations, whereas the Mini-SPIN should be employed with caution due to its lower discriminative performance.

## Conclusion

The present findings provide preliminary support for the continued applicability of the established German SPIN cut-off of 25 in contemporary university students and represent a preliminary replication of previous findings reported by [20]. In contrast, the Mini-SPIN showed lower discriminative accuracy in this sample, suggesting that the full SPIN is preferable in comparable student populations. Exploratory analyses provide preliminary indications that sex-specific cut-offs may warrant further investigation in larger and more balanced samples. Finally, future refinements of the SPIN may benefit from the inclusion of brief assessments of clinically significant distress, functional impairment, and physiological symptoms to enhance its screening precision.

## Supplementary Information

Supplementary Material 1.

## Data Availability

All data generated or analyzed during the current study are available from the corresponding author on reasonable request.

## Abbreviations

AUC: Area under the curve
DSM-5: Diagnostic and Statistical Manual of Mental Disorders, Fifth Edition
FN: False negative
FP: False positive
M: Mean
Mini-SPIN: Mini Social Phobia Inventory
NPV: Negative predictive Value
PPV: Positive predictive value
ROC: Receiver operating characteristic
SAD: Social Anxiety Disorder
SCID-5-CV: Structured Clinical Interview for DSM-5 Disorders, Clinician Version
SD: Standard Deviation
SPIN: Social Phobia Inventory
TN: True negative
TP: True positive
