# Supplementary material for: Preliminary evaluation of the German SPIN and Mini-SPIN for screening social anxiety disorder in university students
Source: BMC Psychol. 2026 Jul 10;14:1030. doi: 10.1186/s40359-026-05119-8 (PMC13352864; doi:10.1186/s40359-026-05119-8)
Supplement: Supplementary file 1 — Supplementary Material 1. [file 40359_2026_5119_MOESM1_ESM.docx]

Supplementary Material

# Supplementary Tables

**Table S1**

Demographic and health characteristics of the sample.

| **Sample characteristics** | **Total (N = 65)** |
| --- | --- |
| **Age** (years) Mean  S.D. Range | 21.02 2.74 18 - 32 |
| **Gender,** n (%) Female Male | 58 (89.2) 7 (10.8) |
| **Highest educational qualification,** n (%) General qualification for university entrance University degree Completed vocational training | 59 (90.8) 4 (6.1) 2 (3.1) |
| **Language level German,** n (%) Native language Level C1 Level C2 | 58 (89.2) 4 (6.2) 3 (4.6) |
| **Neurological disorders,** n (%) Migraine | 2 (3.1) |
| **Self-reported mental disorders,** n (%) Depression  Anorexia nervosa | 3 (4.6) 1 (1.5) |

*Note.* The table displays mean values and standard deviations (S.D.) for metric variables and absolute (*n*) and relative (%) frequencies for categorial variables.

**Table S2**

Cross-tabulation of SPIN-based classification (cut-off ≥ 25), Mini-SPIN based classification (cut-off ≥ 6) and SCID-5 diagnosis of SAD, stratified by sex.

|  | **Female** |  |  | |
| --- | --- | --- | --- | --- |
|  |  |  | **SCID-5** | |
|  | **SPIN** **≥ 25**^a^ |  | *criteria fulfilled* | *criteria not fulfilled* |
|  |  | *cut-off fulfilled* | TP = 8 (13.8%) | FP = 17 (29.3%) |
|  |  | *cut-off not fulfilled* | FN = 1 (1.7%) | TN = 32 (55.2%) |
|  |  |  | **SCID-5** | |
|  | **Mini- SPIN ≥ 6**^b^ |  | *criteria fulfilled* | *criteria not fulfilled* |
|  |  | *cut-off fulfilled* | TP = 5 (8.6%) | FP = 12 (20.7%) |
|  |  | *cut-off not fulfilled* | FN = 4 (6.9%) | TN = 37 (63.8%) |
|  | **Male** |  |  | |
|  |  |  | **SCID-5** | |
|  | **SPIN ≥ 25**^a^ |  | *criteria fulfilled* | *criteria not fulfilled* |
|  |  | *cut-off fulfilled* | TP = 0 (0%) | FP = 0 (0%) |
|  |  | *cut-off not fulfilled* | FN = 1 (14.3%) | TN = 6 (85.7%) |
|  |  |  | **SCID-5** | |
|  | **Mini- SPIN ≥ 6**^b^ |  | *criteria fulfilled* | *criteria not fulfilled* |
|  |  | *cut-off fulfilled* | TP = 0 (0%) | FP = 0 (0%) |
|  |  | *cut-off not fulfilled* | FN = 1 (14.3%) | TN = 6 (85.7%) |

*Note.* ^a^ The table displays the frequency and corresponding percentages (in %) of individuals classified as having or not having SAD according to SPIN and SCID-5, separately by sex (female/male). ^b^ The table displays the frequency and corresponding percentages (in %) of individuals classified as having or not having SAD according to Mini-SPIN and SCID-5, separately by sex (female/male). Frequencies and percentages of concordant (TP, TN), and discordant (FP, FN) classifications are presented. TP = true positive; FP = false positive; TN = true negative; FN = false negative; SPIN = Social Phobia inventory; SCID-5 = Structured Clinical Interview for DSM-5.

**Table S3**Exploratory sex-specific diagnostic indices including Youden-based optimal cut-offs.

| **Measure** | **Sex** | **Optimal Cut-off** | **Youden-Index** | **Sensitivity** | **Specificity** |
| --- | --- | --- | --- | --- | --- |
| SPIN | Female | 24.5 | .542 | 88.9 % | 65.3 % |
| SPIN | Male | 18 * | 1.00 * | 100 % * | 100% * |
| Mini-SPIN | Female | 4.5 | .329 | 77.8 % | 55.1 % |
| Mini-SPIN | Male | 3 * | 1.00* | 100 % * | 100% * |

*Note.* Exploratory Youden-based optimal cut-offs were calculated separately for female and male participants. ** Please note that very restricted number of male participant (see Table S2). Results should not be interpreted and are only presented for completeness.*

# Supplementary Figures


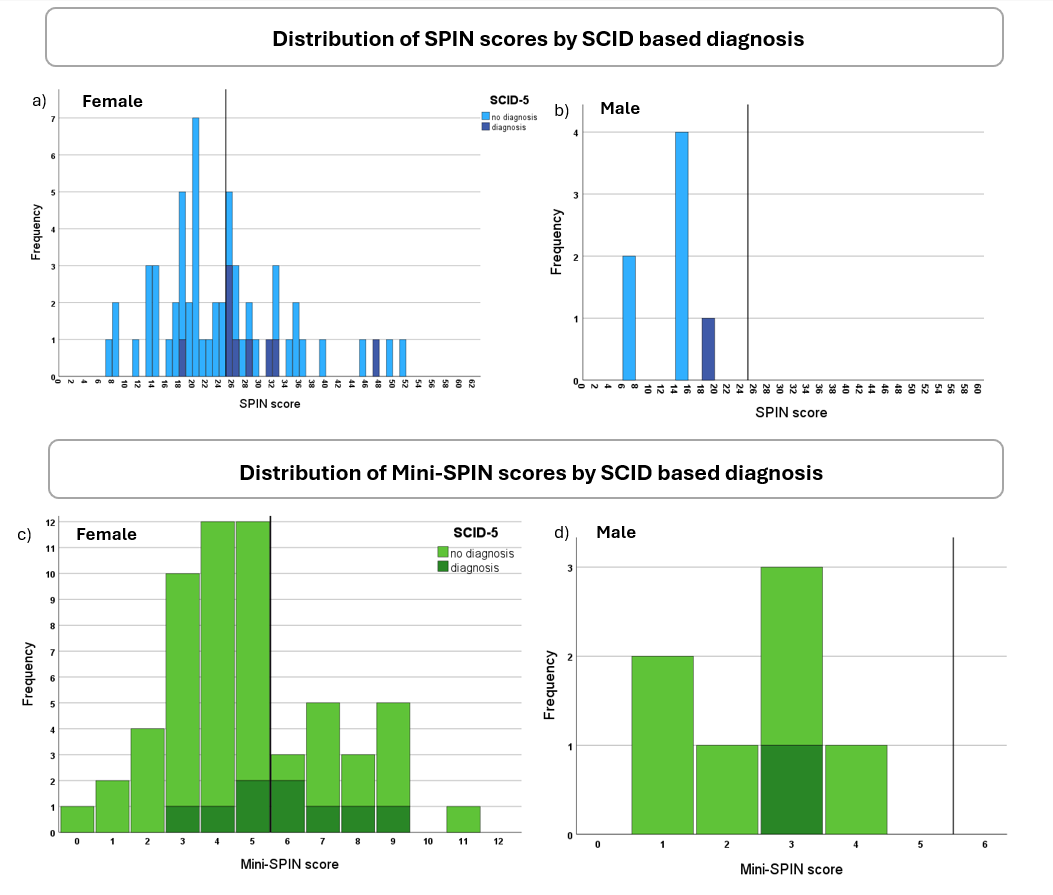


**Figure S1**Histogram depicting the frequency of SPIN and Mini-SPIN scores. Panels show (a) SPIN (female), (b) SPIN (male), (c) Mini-SPIN (female), and (d) Mini-SPIN (male). SPIN panels are shown in blue and Mini-SPIN panels in green. Bars represent the total number of participants per score and are displayed as stacked bars, with shaded segments indicating the number of participants with (darker shades) and without (lighter shades) a SCID-based diagnosis of Social Anxiety Disorder. Vertical black lines indicate the established cut-off scores (SPIN: 25; Mini-SPIN: 6).
